# Identification of the circRNA–miRNA–mRNA network for treating methamphetamine‐induced relapse and behavioral sensitization with cannabidiol

**DOI:** 10.1111/cns.14737

**Published:** 2024-05-03

**Authors:** Liu Liu, Chan Wang, Haowei Wang, Lin Miao, Tong Xie, Yunqing Tian, Xiaodong Li, Yizhen Huang, Xiaofeng Zeng, Bofeng Zhu

**Affiliations:** ^1^ Guangzhou Key Laboratory of Forensic Multi‐Omics for Precision Identification School of Forensic Medicine Southern Medical University Guangzhou Guangdong China; ^2^ NHC Key Laboratory of Drug Addiction Medicine, School of Forensic Medicine Kunming Medical University Kunming Yunnan China

**Keywords:** behavioral sensitization, cannabidiol, competing endogenous RNAs, methamphetamine, relapse

## Abstract

**Aims:**

This study aims to investigate the pharmacological effects and the underlying mechanism of cannabidiol (CBD) on methamphetamine (METH)‐induced relapse and behavioral sensitization in male mice.

**Methods:**

The conditioned place preference (CPP) test with a biased paradigm and open‐field test were used to assess the effects of CBD on METH‐induced relapse and behavioral sensitization in male mice. RNA sequencing and bioinformatics analysis was employed to identify differential expressed (DE) circRNAs, miRNAs, and mRNAs in the nucleus accumbens (NAc) of mice, and the interaction among them was predicted using competing endogenous RNAs (ceRNAs) network analysis.

**Results:**

Chronic administration of CBD (40 mg/kg) during the METH withdrawal phase alleviated METH (2 mg/kg)‐induced CPP reinstatement and behavioral sensitization in mice, as well as mood and cognitive impairments following behavioral sensitization. Furthermore, 42 DEcircRNAs, 11 DEmiRNAs, and 40 DEmRNAs were identified in the NAc of mice. The circMeis2‐miR‐183‐5p‐Kcnj5 network in the NAc of mice is involved in the effects of CBD on METH‐induced CPP reinstatement and behavioral sensitization.

**Conclusions:**

This study constructed the ceRNAs network for the first time, revealing the potential mechanism of CBD in treating METH‐induced CPP reinstatement and behavioral sensitization, thus advancing the application of CBD in METH use disorders.

## Graphical abstract

Cannabidiol (CBD) administration alleviates methamphetamine (METH)‐induced conditioned place preference (CPP) reinstatement and behavioral sensitization in mice. The circMeis2‐miR‐183‐5p‐Kcnj5 network in the nucleus accumbens is involved in this process. This study established the first competing endogenous RNAs network to reveal the potential mechanism of CBD in treating METH‐induced CPP reinstatement and behavioral sensitization, advancing the application of CBD in treating METH use disorders.

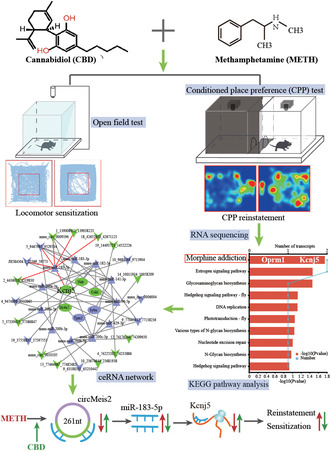

## 1 INTRODUCTION

Drug relapse refers to the recurrence of substance use or addiction symptoms following a period of abstinence, and the high rate of relapse is one of the biggest challenges for the treatment of drug addiction.^1^, ^2^ By the end of 2022, out of the 1.124 million registered drug abusers in China, 1.053 million had a history of relapse.^3^ Methamphetamine (METH) is a widely abused substance globally, specifically emerging as the most commonly abused drug in China.^3^, ^4^ Moreover, it exhibits extraordinarily high mental dependence and relapse rates,^5^ resulting in chronic, intermittent, and recurrent METH use among drug users. Chronic METH use significantly increases the risk of METH‐induced psychosis (MIP), impacting approximately 26%–46% of individuals dependent on METH.^6^ These findings suggest that preventing METH relapse is the most direct approach for treating addiction and reducing the risk of MIP.^7^, ^8^ However, the pathogenesis and therapeutic strategies of METH‐induced relapse remain unclear.

Cannabidiol (CBD) is a non‐psychoactive compound extracted from cannabis sativa, which has potential values for the treatment of METH use disorders. In our previous studies, chronic administration of CBD (40 and 80 mg/kg) via intraperitoneal (IP) injection to rats during acquisition phases or extinction phase attenuates METH (2 mg/kg, IP)‐induced conditioned place preference (CPP).^9^, ^10^ CBD treatment also reduced the motivation of METH self‐administration, drug‐seeking behavior, and METH‐primed relapse in extinguished rats.^11^, ^12^ Additionally, CBD administration during the extinction phase or reinstatement phase of METH‐induced CPP in rats facilitated extinction and suppressed the CPP reinstatement.^13^ Despite these studies preliminary revealing the positive effects of CBD on METH‐induced relapse, the mechanism for the CBD treatment of METH‐induced relapse is poorly understood.

In recent years, non‐coding RNAs (ncRNAs) have become an important biomarker for studying the pathogenesis of disease and identifying drug targets.^14^, ^15^, ^16^, ^17^ ncRNAs, such as miRNAs, silence gene expression by binding to mRNA, thereby affecting the function of organisms. Conversely, the long ncRNAs (lncRNAs) and circular RNAs (circRNAs) can competitively bind miRNAs as competing endogenous RNAs (ceRNAs) to regulate the downstream gene expressions, which plays a positive role in disease treatment.^18^, ^19^ circRNAs are generated through a process called back‐splicing and covalently bonded into a loop structure, which is more stable than linear RNA molecules. They have various biological functions, serving as miRNA sponges, regulators of transcription and splicing, protein traps, and even templates for polypeptide synthesis.^20^ However, no evidence reports the involvement of circRNAs in METH‐induced relapse.

Chronic discontinuous drug exposure leads to drug relapse, and the CPP model is a classic approach used to assess drug use reinstatement.^7^, ^21^ Chronic discontinuous drug exposure also induces behavioral sensitization, which shares common mechanisms with relapse.^22^ Both the sensitization and reinstatement models evaluate the effects of repeated drug exposure on neural function.^23^ Therefore, we assessed the effects of chronic CBD administration on METH‐induced CPP reinstatement and behavioral sensitization. Additionally, we assessed behavioral changes in mice following behavioral sensitization, which encompassed alterations in mood and cognitive function.

The nucleus accumbens (NAc) is a crucial region in the brain's reward system and plays a pivotal role in the development of drug‐induced relapse and behavioral sensitization.^24^, ^25^, ^26^ In this study, we employed high‐throughput sequencing to analyze the expression profiles of circRNA, miRNA, and mRNA in the NAc of mice and constructed a circRNA‐mediated ceRNA network. Our work establishes the first ceRNA network for treating METH‐induced relapse with CBD, potentially advancing the pharmacological development of CBD in METH abuse.

## 2 MATERIALS AND METHODS

### 2.1 Animals

Male C57BL/6J mice, aged 6–8 weeks and weighing 20–24 g, were purchased from Henan Skobes Biotechnology Co., Ltd. The mice were housed in a temperature‐controlled room at 23°C ± 1°C with a humidity level of 45%–55%. They were exposed to a 12‐h light/dark cycle and provided ad libitum access to food and water. The mice were randomly divided into various experimental groups. All procedures followed the guidelines established by the National Institutes of Health for the care and use of laboratory animals and received approval from the Experimental Animal Ethics Committee of Kunming Medical University.

### 2.2 Drug treatment

METH was purchased from the National Institute for Control of Pharmaceutical and Biological Products (Beijing, China) and dissolved in 0.9% NaCl (saline). CBD at 99% purity was purchased from Chengdu Push Bio‐technology Co., Ltd. (Chengdu, China) and was dissolved in a vehicle solution of 5% dimethyl sulfoxide (Solarbio, China) in saline. METH (1 mg/kg or 2 mg/kg), saline (10 mL/kg), and CBD (20 and 40 mg/kg) were administered via IP injection.

### 2.3 Behavioral tests

#### 2.3.1 CPP test

**FIGURE 1:**
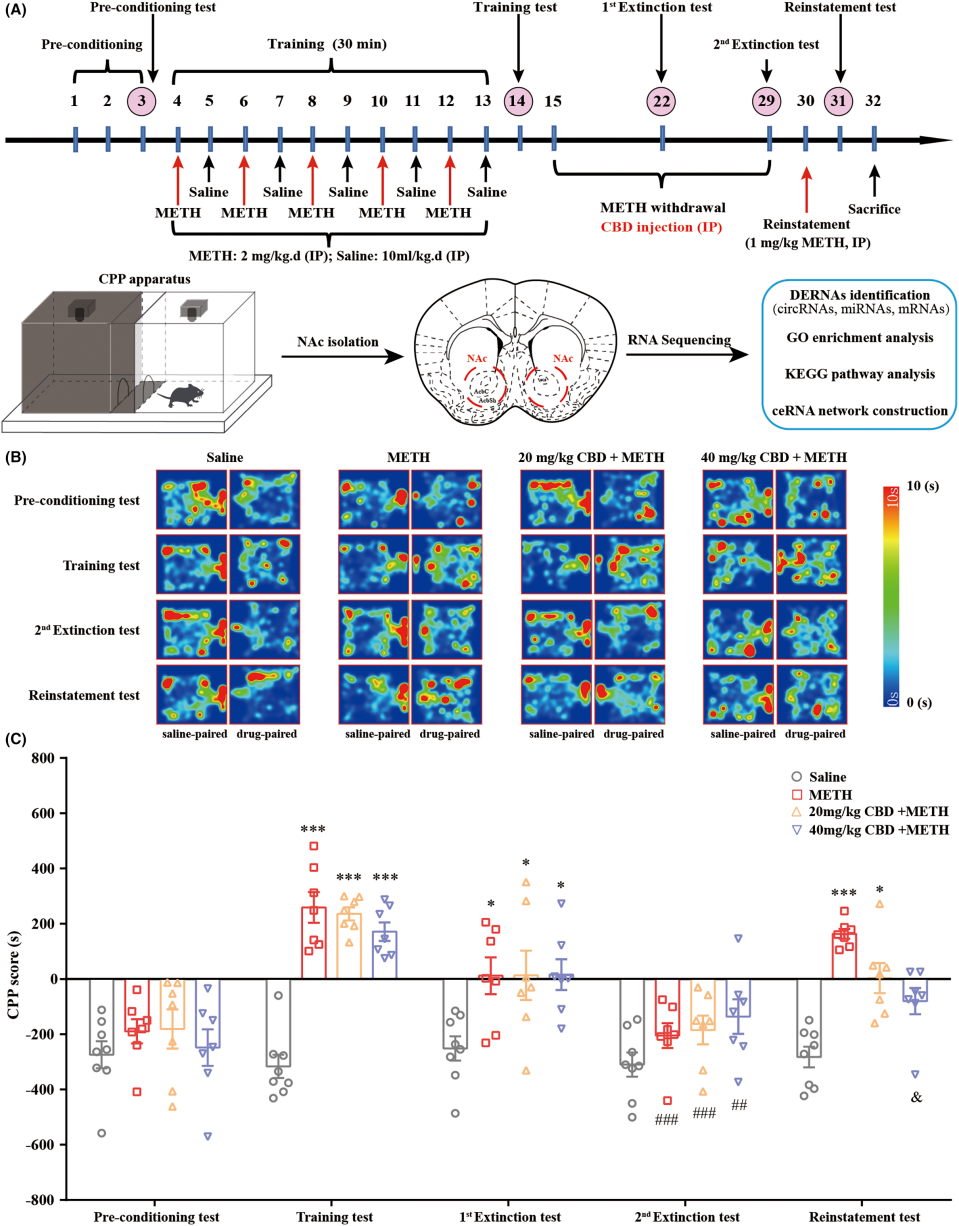
Chronic administration of CBD inhibits METH‐induced CPP reinstatement in mice. (A) CPP test and RNA sequencing protocols. (B) Heat maps of mice's track in the CPP apparatus. (C) Administration of CBD (40 mg/kg, but not 20 mg/kg) during the 14‐day extinction phase inhibited METH‐induced CPP reinstatement in mice. *p < 0.05, ***p < 0.001 versus Pre‐conditioning test; ^##^ *p <* 0.01, ^###^ *p <* 0.001 versus Training test; ^&^ *p <* 0.05 versus METH group. Data were analyzed by two‐way ANOVA followed by Tukey's multiple comparison test, and all values are presented as the mean ± SEM (*n* = 7–8).

The CPP test with a biased paradigm was conducted according to our previous study with modifications,^10^ and the schedule was presented in Figure 1A. Details of the CPP test protocol are described in the Supporting Information.

#### 2.3.2 Behavioral sensitization test

**FIGURE 2:**
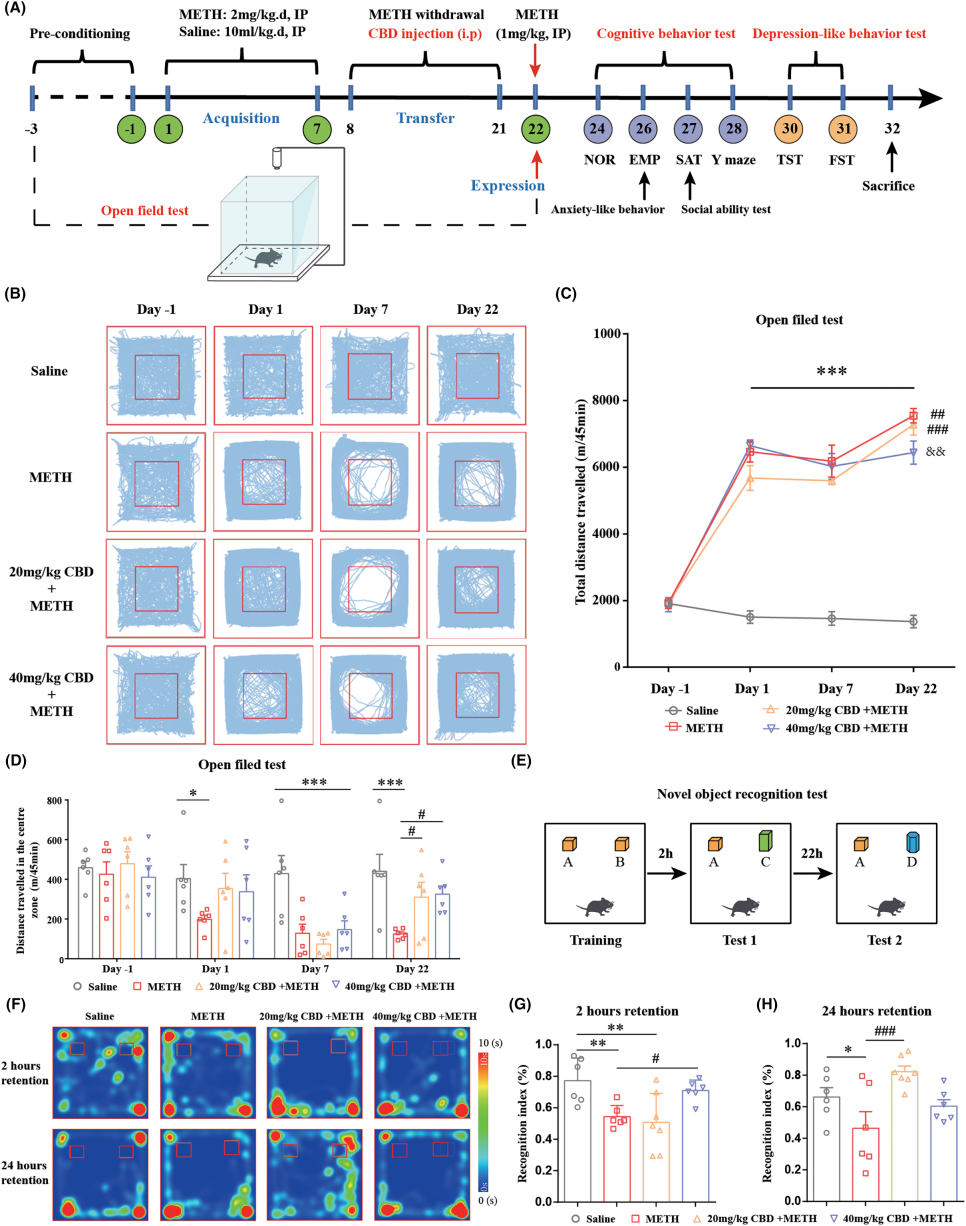
Chronic administration of CBD reduces METH‐induced locomotor sensitization, anxiety‐like behavior, and cognitive impairments in mice. (A) The schedule of locomotor sensitization, mood, and cognitive function tests. (B) The tracks of mice in the open‐field apparatus. (C) Chronic administration of CBD (40 mg/kg, but not 20 mg/kg) reduced METH‐induced locomotor sensitization in mice. ***p < 0.001 versus saline group on the same day; ^##^ *p <* 0.01, ^###^ *p <* 0.001 versus same group on day 7; ^&&^ *p <* 0.01 versus METH group on day 22. (D) Chronic administration of CBD alleviated METH‐induced anxiety‐like behavior in mice. *p < 0.05, ***p < 0.001; ^#^ *p <* 0.05. (E) The protocol of NOR test. (F) Heat maps of mice's track in the NOR test. (G) Chronic administration of CBD (40 mg/kg, but not 20 mg/kg) improved the reduction of recognition index (%) induced by METH after 2 h of training. **p < 0.01; ^#^ *p <* 0.05. (H) Chronic administration of CBD (20 mg/kg, but not 40 mg/kg) improved the reduction of recognition index (%) induced by METH after 24 h of training. *p < 0.05; ^###^ *p <* 0.001. The data were analyzed by two‐way ANOVA (C–D) or one‐way ANOVA (G–H) followed by Tukey's multiple comparison test; all values are presented as the mean ± SEM (*n* = 6–8).

The procedure of the behavioral sensitization test was adapted from previous studies,^6^, ^27^ and the experimental schedule was displayed in Figure 2A. Detailed protocols for behavioral sensitization are described in the Supporting Information.

Additionally, detailed protocols for behavioral testing associated with moods and cognitive function, including novel object recognition (NOR), elevated plus maze (EPM), social ability test (SAT), Y maze test, tail suspension test (TST), and forced swimming test (FST), were displayed in Figure 2A and the Supporting Information.

### 2.4 RNA extraction, library construction and Sequencing

Total RNA was extracted from the NAc of mice for Library construction and Sequencing. Details of these processes are shown in the Supporting Information.

### 2.5 Identification of circRNAs, miRNAs, and mRNAs

Details of these processes are shown in the Supporting Information.

### 2.6 Differentially expressed RNA (DERNAs) identification

We used the DESeq2^28^ package in R software to screen for DEcircRNAs, DEmRNAs, and DEmiRNAs. The significant DEcircRNAs, DEmiRNAs, and DEmRNAs were identified in the comparable group (*p*_adjust_ < 0.05 and |Log2FC| > 1).

### 2.7 GO and KEGG pathway analyses

GO and KEGG pathway analyses were performed using the Goatools and KOBAS^29^ to explore the functions and biological processes of DEmRNAs. The *p* < 0.05 was considered significant, and results were visualized by an online platform (http://www.bioinformatics.com.cn).

### 2.8 ceRNA network construction

To investigate the targeting relationships among the DERNAs, the DEmiRNAs dataset served as the core to predict the target RNAs using miRanda,^30^ based on the DEcircRNAs and DEmRNAs dataset. Subsequently, a ceRNA network was visualized to reveal the competitive binding of miRNAs by circRNAs and mRNAs using Cytoscape 3.7.1 software. All of the bioinformatic analyses were performed on the online platform of Majorbio Cloud Platform (https://cloud.majorbio.com/).^31^

### 2.9 RNA extraction and qRT–PCR

Total RNA was extracted from the NAc of mouse brains using the TransZol Up Plus RNA Kit (TransGen Biotech, China) according to the manufacturer's protocol. qRT–PCR assays were performed using the CFX96 RT‐PCR system (Bio‐Rad, USA). The protocols for RNA quantification are presented in the Supporting Information, and the primer sequences used in qRT–PCR are listed in Table S1.

### 2.10 Western blot

The total protein of NAc was isolated by treating it with ice‐cold RIPA buffer containing protease inhibitors. Detailed procedure of the western blot is presented in the Supporting Information.

### 2.11 Statistical analysis

All the statistical analyses were performed using GraphPad Prism 6.02 (GraphPad Software, USA). The Shapiro–Wilk test was used to assess the normality of the data distribution. One‐way or two‐way analysis of variance (ANOVA) followed by Tukey's multiple comparison test was performed for comparison among multiple groups. All data were expressed as mean ± standard error (SEM), and *p* < 0.05 was considered statistically significant. Details of the statistical analyses applied to each data set, including the number of subjects and the corresponding *p*‐values, are described in the figure legends.

## 3 RESULTS

### 3.1 Chronic CBD administration inhibits reinstatement of METH‐induced CPP in extinguished mice

To record the tracks of mice in the CPP apparatus, heat maps were generated based on the time spent by mice using the Visutrack system (Figure 1B). After 10 days of training, 2 mg/kg METH successfully induced a significant CPP in mice compared with the pre‐conditioning test (Figure 1C). The data from the first Extinction test showed that neither the saline nor the CBD treatment group exhibited a significant decrease in CPP scores after 7‐day CPP extinction session compared to the Training test (Figure 1C). After 14‐day CPP extinction session, the CPP scores of the second Extinction test were significantly decreased in both saline and CBD treatment groups compared to the Training test (Figure 1C). This implies that CBD has no positive effects on CPP extinction in mice. Subsequently, the mice were challenged with a dose of 1 mg/kg METH in the drug‐paired compartment and confined them for 30 min (day 30). The data from the Reinstatement test (day 31) suggested that 1 mg/kg METH induced significant reinstatement of CPP in extinguished mice (Figure 1C). Notably, the administration of 40 mg/kg CBD to mice markedly inhibited the reinstatement of METH‐induced CPP (Figure 1C).

### 3.2 Chronic CBD administration inhibits METH‐induced behavioral sensitization in mice

Figure 2B shows the tracks of mice in the open‐field apparatus. On day 1 of testing, the administration of 2 mg/kg METH significantly increased the total distance traveled by mice in the open‐field apparatus compared to the saline group (Figure 2C). After 7 days of repeated METH administration, there was no obvious change in the total distance traveled by mice compared to the day 1 test. Subsequently, the mice underwent METH withdrawal and CBD treatment for 14 days. On day 22, the mice challenged with a dose of 1 mg/kg METH showed a significant increase in the total distance traveled in the open‐field apparatus (Figure 2C), indicating successful behavioral sensitization in mice by METH. Additionally, treatment with 20 mg/kg CBD during the METH withdrawal phase failed to regulate METH‐induced behavioral sensitization in mice. However, the administration of 40 mg/kg CBD significantly suppressed METH‐induced behavioral sensitization in mice (Figure 2C).

### 3.3 Chronic CBD administration alleviates METH‐induced moods and cognitive impairments in mice

To comprehensively examine the effects of CBD on abnormal behavioral induced by METH in mice, we evaluated cognitive function, anxiety‐like behavior, and depression‐like behavior. In the OFT test, repeated METH exposure significantly reduced the distance traveled by mice in the central area (Figure 2D), suggesting that METH exposure produced significant anxiety‐like behavior in mice. However, treatment with both 20 mg/kg and 40 mg/kg CBD effectively reversed this effect. The NOR test protocol is shown in Figure 2E, with mouse tracks depicted in Figure 2F. After 2 h of training, administration of 40 mg/kg CBD rescued the METH‐induced decrease of recognition index in mice (Figure 2G). After 24 h of training, administration of 20 mg/kg CBD, but not 40 mg/kg CBD, significantly improved the recognition index of mice compared to the METH group (Figure 2H).

**FIGURE 3:**
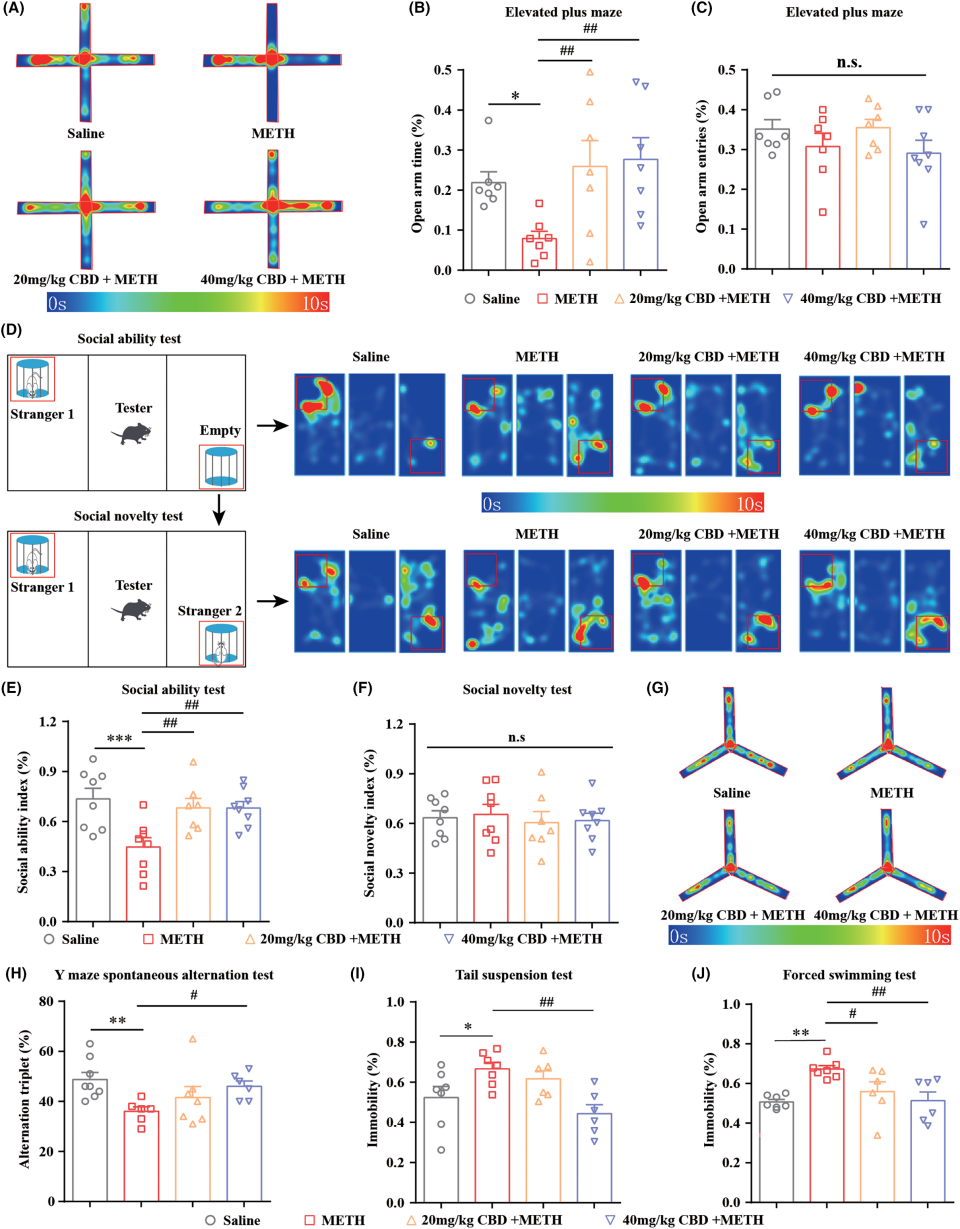
Chronic administration of CBD alleviates METH‐induced moods and cognitive impairments in mice. (A) Heat map of mice's track in the EPM apparatus. (B) Chronic administration of CBD increased the time spent in the open arms in METH‐treated mice. *p < 0.05; ^##^ *p <* 0.01. (C) Both CBD and METH treatment do not affect the number of entries into the open arms in mice. n.s: not significant. (D) Heat maps of mice's track in the three‐chamber apparatus. (E) Chronic administration of CBD improved the reduction of the social ability index (%) caused by METH in mice. ***p < 0.001; ^##^ *p <* 0.01. (F) Both CBD and METH treatment do not affect the social novelty index (%) in mice. n.s, not significant. (G) Heat map of mice's track in the Y maze apparatus. (H) Chronic administration of CBD (40 mg/kg, but not 20 mg/kg) rescued the reduction of the alteration triplet (%) induced by METH in mice. **p < 0.01; ^#^ *p <* 0.05. (I) Chronic administration of CBD (40 mg/kg, but not 20 mg/kg) decreased the immobility time of METH‐treated mice in the TST test. *p < 0.05; ^##^ *p <* 0.01. (J) Chronic administration of CBD decreased the immobility time of METH‐treated mice in the FST test. **p < 0.01; ^#^ *p <* 0.05, ^##^ *p <* 0.01. All data were analyzed by one‐way ANOVA followed by Tukey's multiple comparison test; all values are presented as the mean ± SEM; *n* = 6–8.

In the EPM test, METH exposure reduced the time spent by mice in the open arms, indicating that METH induced anxiety‐like behavior in mice (Figure 3A,B). These effects were alleviated by the chronic CBD administration. No effect on open arms entries was observed (Figure 3C). In the three‐chamber test, repeated METH exposure significantly impaired the social ability of mice, which was blocked by chronic CBD treatment (Figure 3D,E). No impact was observed in the social novelty test (Figure 3F). In the Y maze test, CBD treatment reversed the METH‐induced reduction in alternation triplet, suggesting that the CBD improved the METH‐induced cognitive impairment in mice (Figure 3G,H). In the TST and FST tests, CBD effectively attenuated the depression‐like behavior in mice following METH exposure (Figure 3I,J).

### 3.4 RNA sequencing reveals the circRNAs expression profile in NAc of CPP mice

**FIGURE 4:**
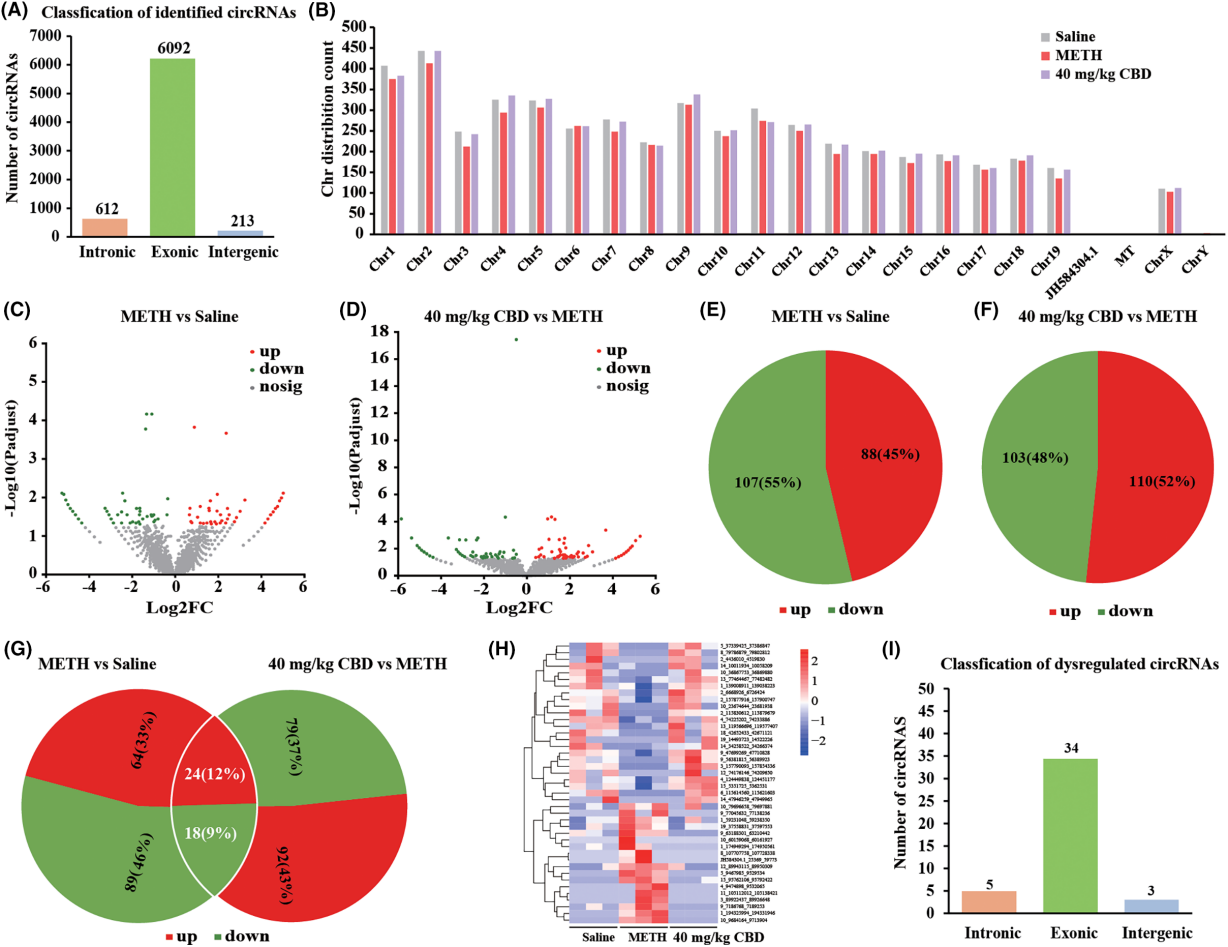
circRNAs profiling in the NAc of mice from saline, METH, and 40 mg/kg CBD + METH groups. (A) The number of identified circRNAs and their composition in terms of gene distribution. (B) The distribution of identified circRNAs on the mouse chromosomes. (C) The volcano plot of DEcircRNAs in the NAc between the saline‐treated mice and METH‐treated mice. (D) The volcano plot of DEcircRNAs in the NAc between the 40 mg/kg CBD + METH‐treated mice and METH‐treated mice. (E) The number of upregulated and downregulated circRNAs in METH‐treated mice compared to the saline‐treated mice. (F) The number of upregulated and downregulated circRNAs in 40 mg/kg CBD + METH‐treated mice compared to the METH‐treated mice. (G) There are a total of 42 shared DEcircRNAs among (E) and (F). (H) A heatmap displayed the expression of 42 shared circRNAs in three paired samples of NAc from saline‐treated mice, METH‐treated mice, and 40 mg/kg CBD + METH‐treated mice. (I) The composition of 42 shared DEcircRNAs in terms of gene distribution.

To screen the molecular targets for CBD treatment of METH‐induced CPP reinstatement in mice, we performed RNA sequencing analysis to explore the differentially expressed genes (DEGs) between the saline, METH, and 40 mg/kg CBD treatment groups. A total of 6917 circRNAs were identified from these groups. Figure 4A illustrates their distribution based on gene composition, and Figure 4B displays their chromosomal distribution in mice. The volcano plots in Figure 4C,D showed the DEcircRNAs that were significantly altered in the comparable groups. We identified 195 DEcircRNAs in METH‐treated mice compared to saline‐treated mice, with 88 upregulated and 107 downregulated circRNAs (Figure 4E). Moreover, we detected 213 DEcircRNAs in 40 mg/kg CBD‐treated mice compared to METH‐treated mice, with 110 upregulated and 103 downregulated circRNAs (Figure 4F). Notably, 24 circRNAs were upregulated in METH‐treated mice but downregulated in CBD‐treated mice, while 18 circRNAs were downregulated in METH‐treated mice but upregulated in CBD‐treated mice (Figure 4G and Table S2). A heatmap was created to visualize the expression of these DEcircRNAs in different samples (Figure 4H and Table S2), and Figure 4I depicts the gene composition‐based distribution of these circRNAs.

### 3.5 RNA sequencing reveals the mRNAs' expression profile in NAc of CPP mice

**FIGURE 5:**
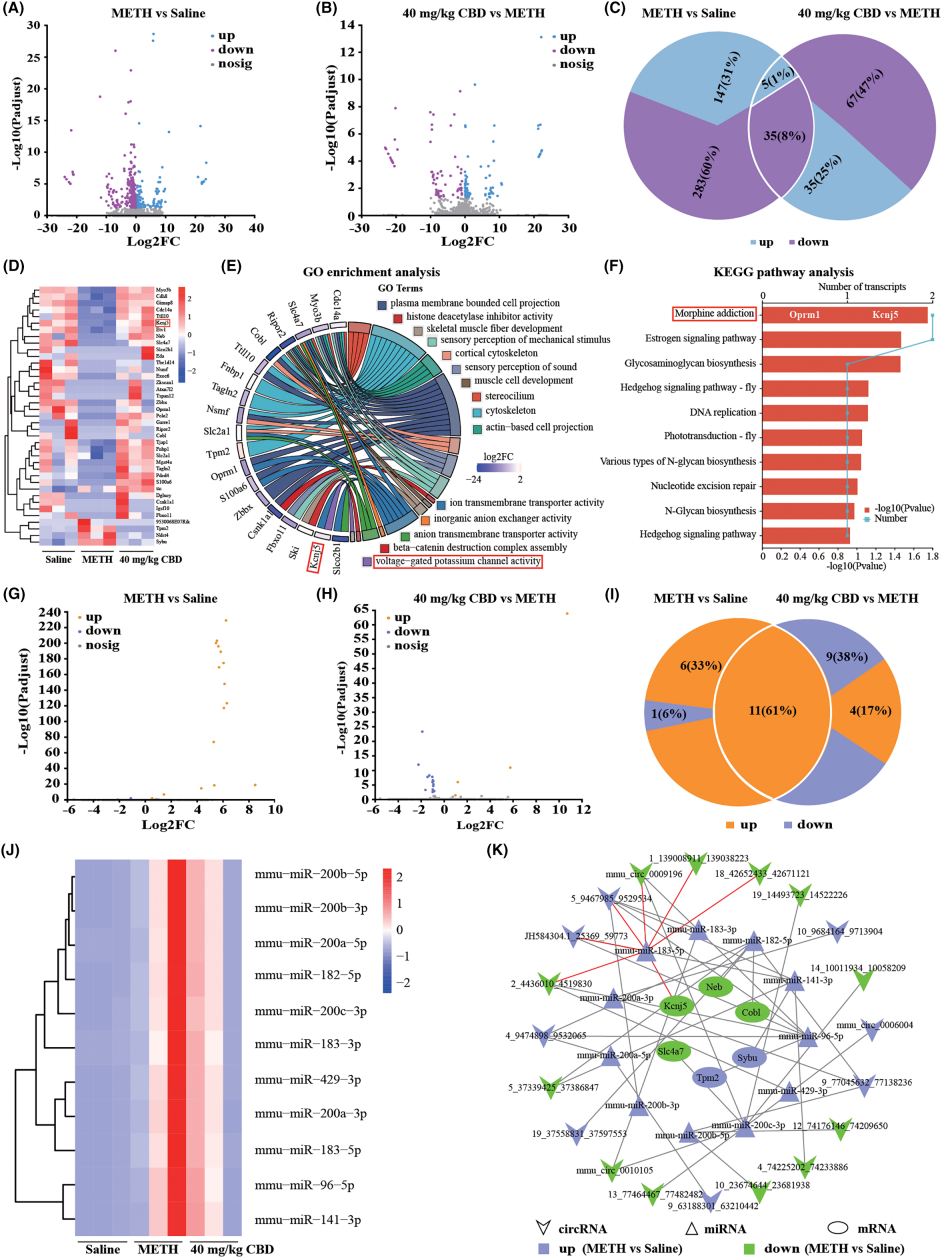
mRNAs and miRNAs profiling in the NAc of mice from saline, METH, and 40 mg/kg CBD + METH groups. (A) The volcano plot of DEmRNAs in the NAc between the saline‐treated mice and METH‐treated mice. (B) The volcano plot of DEmRNAs in the NAc between the 40 mg/kg CBD + METH‐treated mice and METH‐treated mice. (C) There is a total of 40 shared DEmRNAs among the three groups. (D) A heatmap displayed the expression of 40 shared mRNAs in three paired samples of NAc from saline‐treated mice, METH‐treated mice, and 40 mg/kg CBD + METH‐treated mice. (E) The top 15 terms in the GO enrichment analysis of 40 shared DEmRNAs. (F) The top 10 pathways in the KEGG pathway analysis of 40 shared DEmRNAs. (G) The volcano plot of DEmiRNAs in the NAc between the saline‐treated mice and METH‐treated mice. (H) The volcano plot of DEmiRNAs in the NAc between the 40 mg/kg CBD + METH‐treated mice and METH‐treated mice. (I) There are a total of 11 shared DEmiRNAs among the three groups. (J) A heatmap displayed the expression of 11 shared miRNAs in three paired samples of NAc from saline‐treated mice, METH‐treated mice, and 40 mg/kg CBD + METH‐treated mice. (K) ceRNA network that was constructed based on the dataset of shared 42 DEcircRNAs, 11 DEmiRNAs, and 40 DEmRNAs using miRanda.

The volcano plots in Figure 5A,B present the DEmRNAs significantly altered in the comparable groups. A total of 470 DEmRNAs were identified in METH‐treated mice compared to saline‐treated mice, with 152 mRNAs upregulated and 318 mRNAs downregulated in METH‐treated mice (Figure 5C). In CBD‐treated mice, 71 mRNAs were upregulated, and 71 mRNAs were downregulated compared to METH‐treated mice (Figure 5C). Importantly, 35 mRNAs were downregulated in METH‐treated mice but upregulated in CBD‐treated mice, while 4 mRNAs were upregulated in METH‐treated mice but downregulated in CBD‐treated mice. There was 1 mRNA that was upregulated in both METH‐treated mice and CBD‐treated mice (Figure 5C and Table S3). A heat map was generated to visualize the expression of these DEmRNAs in different samples (Figure 5D and Table S3).

### 3.6 GO enrichment and KEGG pathway analyses for the 40 DEmRNAs

The GO enrichment analysis, respectively, revealed the top 5 terms in cellular components, biological processes, and molecular functions, and identified 19 DEmRNAs associated with these terms (Figure 5E and Table S4). Previous studies have reported the significant involvement of voltage‐gated potassium channels in mediating the rewarding effects of substance abuse.^32^, ^33^ *KCNJ5* gene encodes the protein of G protein‐activated inward rectifier potassium channel 4 (GIRK4). GIRK channels are expressed in brain regions closely associated with drug dependence, mediating slow inhibitory postsynaptic currents (IPSCs) and regulating the excitability of dopamine (DA) neurons.^34^ Nonetheless, there is currently no evidence supporting the involvement of GIRK4 in the reinstatement of METH‐induced CPP in mice. The KEGG pathway analysis identified significant enrichment of the *KCNJ5* and *OPRM1* genes in the pathway associated with morphine addiction (Figure 5F and Table S5). This finding lends further support to the potential targeting of the *KCNJ5* gene and its encoded protein GIRK4 for CBD treatment of METH‐induced CPP reinstatement in mice.

### 3.7 RNA sequencing reveals the miRNA expression profile in NAc of CPP mice

The volcano plots in Figure 5G,H displayed the DEmiRNAs that were significantly altered in the comparable groups. A total of 18 DEmiRNAs were detected in METH‐treated mice compared to saline‐treated mice, 17 miRNAs were upregulated and 1 miRNA was downregulated among these miRNAs. In CBD‐treated mice, 4 miRNAs were upregulated and 17 miRNAs were downregulated compared to METH‐treated mice. Notably, 11 miRNAs were upregulated in the METH‐treated mice but downregulated in the CBD‐treated mice (Figure 5I). A heat map was generated to illustrate the expression of these miRNAs in different samples (Figure 5J and Table S6).

### 3.8 ceRNA network construction for the CBD treatment of METH‐induced CPP reinstatement in mice

To reveal the interaction among the 42 DEcircRNAs, 40 DEmRNAs, and 11 DEmiRNAs, a ceRNA network was constructed using miRanda and visualized with Cytoscape software (Figure 5K and Table S7). The ceRNA network comprised 20 circRNAs, 11 miRNAs, and 6 mRNAs. Among these RNAs, 8 circRNAs, 11 miRNAs, and 2 mRNAs were upregulated in METH‐treated mice but downregulated in CBD‐treated mice. Conversely, 12 circRNAs and 4 mRNAs were downregulated in METH‐treated mice but upregulated in CBD‐treated mice. Based on the results of GO enrichment and KEGG pathway analysis, we focused on the RNAs that interacted with Kcnj5. The Kcnj5 can interact with mmu‐miR‐183‐5p, which could in turn interact with 6 circRNAs. Moreover, an inverse relationship was observed between the expression of Kcnj5 and miR‐183‐5p, suggesting that miR‐183‐5p may silence Kcnj5 expression by competitively binding to it. The expression trends of 4 circRNAs, 2_4436010_4519830 (termed circFrmd4a), mmu_circ_0009196 (termed circMeis2), 1_139008911_139038223 (termed circDennd1b), and 18_42652433_42671121 (termed circTcerg1), are consistent with Kcnj5 expression but opposite to that of miR‐183‐5p. This suggests that these circRNAs may reverse the silencing effect on Kcnj5 expression by competitively binding with miR‐183‐5p.

### 3.9 Validation of the expression of ceRNAs in the CPP and behavioral sensitization models of mice

**FIGURE 6:**
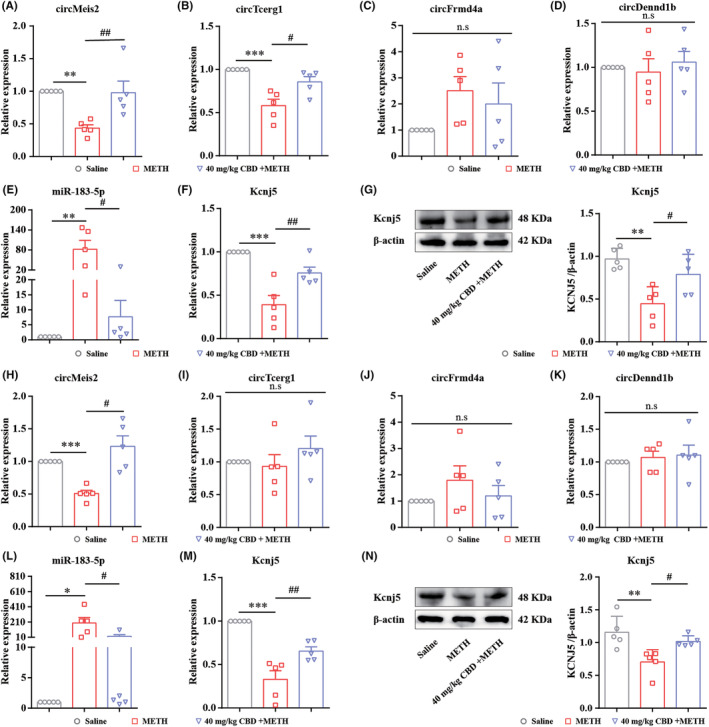
circMeis2‐miR‐183‐5p‐Kcnj5 network involves the positive effects of CBD on treating METH‐induced CPP reinstatement and behavioral sensitization in mice. (A–D) circRNAs expression in the NAc of CPP mice. (E) miR‐183‐5p expression in the NAc of CPP mice. (F,G) Kcnj5 expression in the NAc of CPP mice. (H–K) circRNAs expression in the NAc of behavioral sensitized mice. (L) miR‐183‐5p expression in the NAc of behavioral sensitized mice. (M,N) Kcnj5 expression in the NAc of behavioral sensitized mice. *p < 0.05, **p < 0.01, ***p < 0.001; ^#^ *p <* 0.05, ^##^ *p <* 0.01. All data were analyzed by one‐way ANOVA followed by Tukey's multiple comparison test; all values are presented as the mean ± SEM (*n* = 5).

In the CPP model, circMeis2 and circTcerg1 were significantly downregulated in METH‐treated mice but rescued in 40 mg/kg CBD‐treated mice (Figure 6A,B), consistent with the results of RNA sequencing. However, circFrmd4a (Figure 6C) and circDennd1b (Figure 6D) showed no significant change in METH or CBD‐treated mice. The expression of miR‐183‐5p was dramatically upregulated in METH‐treated mice but recovered in CBD‐treated mice (Figure 6E). Kcnj5 mRNA and protein were significantly reduced in METH‐treated mice but rescued in CBD‐treated mice (Figure 6F,G). In the behavioral sensitization model, circMeis2 (Figure 6H) and Kcnj5 (Figure 6M,N) were downregulated in METH‐treated mice but rescued in CBD‐treated mice, consistent with the CPP model. The expressions of circTcerg1 (Figure 6I), circFrmd4a (Figure 6J), and circDennd1b (Figure 6K) showed no significant changes. The miR‐183‐5p expression was markedly upregulated in METH‐treated mice but suppressed in CBD‐treated mice (Figure 6L). In summary, these findings suggest that the circMeis2‐miR‐183‐5p‐Kcnj5 network involves in the positive effects of CBD on METH‐induced CPP reinstatement and behavioral sensitization.

## 4 DISCUSSION

In the present study, repeated METH administration successfully induced a significant CPP (using a biased place conditioning paradigm) in male mice, consistent with our previous findings in male rats.^9^, ^10^ However, the duration of extinction days varies in METH‐induced CPP studies, ranging from 5 to 7 days^35^, ^36^, ^37^ to long periods.^11^, ^38^ In our previous study, a 10‐day extinction period failed to extinguish the METH‐induced CPP.^10^ As reported in a previous study, the 11‐day extinction had no significant impact on METH‐induced CPP.^39^ In this study, a significant extinction of METH‐induced CPP was observed after a 14‐day extinction period but not after a 7‐day extinction period. Therefore, the extinction duration can differ across various studies, experimental designs, and animal models, emphasizing the need for appropriate CPP testing intervals. Based on these findings, the duration of the extinction phase should be adjusted accordingly, either shortened or lengthened.

Behavioral sensitization is characterized by the progressive and enduring enhancement of behavioral and neurological responses to repeated, intermittent exposure to a stimulus, such as a drug or stressor. Re‐exposure to METH induced behavioral sensitization, which may enhance the motivation of drug seeking/taking to trigger CPP reinstatement.^40^ Additionally, behavioral sensitization caused by chronic drug use shares common mechanisms with drug relapse. First, mice administered with the same method of cocaine training that triggers reinstatement of cocaine‐seeking also showed locomotor sensitization.^41^ Furthermore, the sensitization and reinstatement induced by drug abuse involve shared circuitry, neurotransmitter systems, and receptors.^23^ In the present study, we identified a novel ceRNA mechanism, the circMeis2‐miR‐183‐5p‐Kcnj5 network, which is involved in both CPP reinstatement and METH‐induced behavioral sensitization. Despite the observed overlapping mechanisms in drug reinstatement and sensitization, the specific role of sensitization in drug reinstatement remains undefined. An indicator is that a single challenge dose of METH (1 mg/kg, IP) successfully reinstated METH‐induced CPP in extinguished mice.

In recent years, CBD has been studied in the field of substance use disorders, such as METH addiction. Our previous study demonstrated that administration of CBD (40 and 80 mg/kg, but not 10 and 20 mg/kg) in the acquisition or extinction phase of CPP effectively reduced METH‐induced CPP and facilitated CPP extinction in male rats.^9^, ^10^ In this study, we further explored the effects of CBD (20 and 40 mg/kg) on METH‐induced CPP reinstatement and behavioral sensitization in male mice. We found that repeated administration of CBD (40 mg/kg, but not 20 mg/kg) during the METH withdrawal period significantly inhibits METH‐induced CPP reinstatement and behavioral sensitization in mice. However, in this study, the administration of CBD had no discernible effects on CPP extinction in mice, which differs from our prior investigation as well as other existing studies.^13^ Species differences among experimental subjects may be one of the reasons for the observed discrepancies. For instance, administering CBD (40 and 80 mg/kg) for 10 consecutive days during extinction phase facilitated CPP extinction in rats.^10^ However, in the present study, mice treated with CBD (20 and 40 mg/kg) for extended periods during the extinction phase did not exhibit such a facilitating effect. The method of CBD administration also contributes to this discrepancy. Administering CBD (10 and 50 μg/5 μL) via intracerebroventricular (ICV) reduced the extinction latency of CPP,^13^ yet this effect was not observed with CBD administration via IP in the current study. In a study of cocaine self‐administration, CBD (10 and 20 mg/kg, IP) also demonstrated no impact on rat extinction,^42^ aligning with the findings of our study. Additionally, age, psychological factors, and environmental enrichment (EE) significantly influenced CPP extinction. Adolescent rats exhibit delayed CPP extinction compared to adult rats.^43^, ^44^ Stress exposure increased anxiety‐like behavior in mice, delaying CPP extinction, while EE significantly reduced the duration of stress‐induced delay in extinction^45^ and cocaine‐seeking behavior during the extinction phase.^46^

Furthermore, it should be noted that the effective dosage of CBD for treating METH‐induced CPP reinstatement can vary across studies, depending on factors such as the animal models used and the manner of CBD administration. In this study, the administration of CBD (40 mg/kg, but not 20 mg/kg) via IP injection effectively inhibited METH‐induced CPP reinstatement and behavioral sensitization in mice. In the rats with METH self‐administration, CBD treatment (80 mg/kg, but not 40 mg/kg, or 20 mg/kg) via IP injection attenuated METH‐primed relapse and METH‐seeking behavior in extinguished rats.^12^ A previous study showed that a single dose of CBD (10 μg/5 μL) administration via intracerebroventricular (ICV) injection could suppress the reinstatement of METH‐induced CPP in extinguished rats.^47^ It would be worthwhile to investigate whether a single dose of CBD at 40 mg/kg via IP administration is effective in treating CPP reinstatement and behavioral sensitization.

D1‐like and D2‐like dopamine receptors, which are classical receptors within the reward system, play a crucial role in mediating the anti‐drug addiction effects of CBD. CBD inhibits METH‐induced CPP by interacting with D1 and D2 dopamine receptors in the hippocampus (HIP)^48^, ^49^ and NAc.^50^ Our previous studies have identified that the Sigma1 receptor in the NAc, HIP, prefrontal cortex (PFC), and ventral tegmental (VTA) regions was involved in the inhibitory effects of CBD on METH‐induced CPP.^9^, ^10^ As a natural anti‐inflammatory compound, CBD effectively prevents stress‐ and drug‐induced reinstatement of METH in extinguished rats by modulating the expression of neuroinflammatory factors.^11^ Additionally, the cannabinoid type 1 receptor (CB1R), serotonergic receptor 1A (5‐HT1A), transient receptor potential V1 (TRPV1) channels, and peroxisome proliferator‐activated receptor (PPAR‐γ) are potential molecular targets for CBD in treating drug addiction.^51^ However, their specific roles in treating METH addiction with CBD need further elucidation.

The present study revealed a novel ncRNA‐mediated mechanism, the circMeis2‐miR‐183‐5p‐Kcnj5 network, involved in the CBD treatment of METH‐induced CPP reinstatement and behavioral sensitization in mice. circMeis2 (mmu_circ_ 0009196) originates from the host gene *MEIS2* on chromosome 2: 115830612–115,879,679 (261 nt). It consists of two exons (exons 7–8) from the *MEIS2* gene locus. *MEIS2* encodes a transcription factor that is part of the highly conserved triamino acid loop extension (TALE) homeobox protein family.^52^ *MEIS2* is crucial for cell fate determination, organogenesis, and functional maintenance during embryonic development.^53^, ^54^, ^55^ Notably, deletion of Meis2 in mice can block the differentiation and reduce the number of striatal D1 and D2 medium‐sized spiny neurons (MSNs) in the striatum, potentially affecting their behavioral and motor functions.^56^ The plasticity of NAc D1‐MSNs and D2‐MSNs is linked to environmental memory and addictive behavior in drug addiction, influenced by drugs including METH,^57^ morphine,^58^, ^59^ and cocaine.^60^, ^61^ These studies suggest that Meis2 may affect memory and behaviors associated with drug addiction by modulating NAc D1‐MSNs and D2‐MSNs plasticity. However, the role of Meis2 in the process of drug addiction remains unclear as no study has directly addressed this.

miR‐183‐5p is a mature RNA derived from the pre‐miRNA miR‐183, measuring 22 nt in length. Repeated METH injection significantly increased the locomotor activity in rats and upregulated miR‐183‐5p in the striatum.^17^ Our study revealed that the upregulation of miR‐183‐5p in the mice's NAc is involved in the METH‐induced CPP reinstatement and behavioral sensitization. *KCNJ5* gene and its encoded GIRK4 protein are associated with voltage‐gated potassium channel activity and morphine addiction. Although direct evidence supporting the role of GIRK4 in drug addiction is lacking, its family members, GIRK1, GIRK2, and GIRK3, have been found to regulate the mesolimbic reward system associated with cocaine and morphine by mediating the excitability and neurotransmission of dopamine neurons.^62^, ^63^ Moreover, the *weaver* mutant mice present abnormal GIRK2 function, leading to a significant reduction in METH‐induced CPP and dopamine release in the NAc.^64^ These findings suggest that GIRK4 may represent a novel target for treating METH addiction, as well as METH‐induced CPP reinstatement and behavioral sensitization.

## 5 CONCLUSIONS

Our study demonstrated that chronic administration of CBD (40 mg/kg, IP) during the METH withdrawal phase effectively inhibits CPP reinstatement and behavioral sensitization in mice. By employing RNA sequencing and bioinformatic analyses, we constructed the first ceRNA network in the NAc for treating METH‐induced CPP reinstatement with CBD. Subsequently, it was proven that the circMeis2‐miR‐183‐5p‐Kcnj5 network is involved in the positive effects of CBD for treating METH‐induced CPP reinstatement and behavioral sensitization, providing evidence that supports the existence of the common mechanism between drug relapse and sensitization. According to the ceRNA theory, circMeis2 potentially regulates the expression and function of Kcnj5 by acting as a molecular sponge for miR‐183‐5p. Future studies will investigate the role of the circMeis2‐miR‐183‐5p‐Kcnj5 network in regulating METH‐induced CPP reinstatement and behavioral sensitization in mice using chemical genetics methods. Additionally, it would be valuable to explore the neuron‐specific mechanism of the circMeis2‐miR‐183‐5p‐Kcnj5 network in the NAc following repeated METH exposure.

## CONFLICT OF INTEREST STATEMENT

The authors declare no conflicts of interest.

## Supporting information


TableS1

TableS2

TableS3

TableS4

TableS5

TableS6

TableS7

DataS1

DataS2

## Data Availability

The data used to support the findings of this study are available from the corresponding author upon request.
