# Supplementary material for: Identification of the circRNA–miRNA–mRNA network for treating methamphetamine‐induced relapse and behavioral sensitization with cannabidiol
Source: CNS Neurosci Ther. 2024 May 3;30(5):e14737. doi: 10.1111/cns.14737 (PMC11069028; doi:10.1111/cns.14737)
Supplement: Supplementary file 8 — DataS1 [file CNS-30-e14737-s001.docx]

**Supplementary Materials and Methods**

**Conditioned place preference (CPP) test**

The CPP apparatus used in this study consisted of three compartments (Xinruan Information Technology Co., Ltd., China). One compartment had a grid floor and black walls (20 × 20 × 20 cm). Another compartment of the same size had a meshed floor and white walls. The middle compartment, which had a flat gray floor and gray walls (5 × 20 × 20 cm), was the initial compartment and connected to the other two compartments via removable doors. The protocol of the CPP test was presented in Figure 1A, and detailed information is available in our previous study^1^. Briefly, each mouse went through four experimental phases: the preconditioning phase (2 days), the training phase (10 days), the extinction phase (14 days), and the reinstatement phase (1 day). The time spent by mice in the apparatus was recorded for 15 minutes on the day following each phase using the VisuTrack system (Xinruan Information Technology Co., Ltd., China). The compartment where mice spent a longer period after the preconditioning phase was defined as the saline-paired compartment, while another compartment was named the drug-paired compartment. During the training phase, the mice received either saline or METH (2 mg/kg, once a day) in either the saline or drug-paired compartment and were subsequently confined to the respective compartment for 30 minutes. During the extinction phase, the mice were injected with either saline or CBD (once a day) and were confined to the drug-paired compartment for 30 minutes. During the reinstatement phase, the mice were administered a challenge dose of METH (1 mg/kg, once a day) and were immediately confined to the drug-paired compartment for 30 minutes. The CPP score was calculated by subtracting the time spent in the saline-paired compartment from the time spent in the drug-paired compartment ^2,3^.

**Behavioral sensitization test**

The behavioral sensitization test was performed in a white chamber (45 cm × 45 cm × 45 cm). The procedure of behavioral sensitization test was referenced in previous studies with modifications ^2,4^, and the schedule was displayed in Figure 2A. Each mouse also went through four experimental phases: the preconditioning phase (3 days), the acquisition phase (7 days), the METH withdrawal phase (14 days), and the expression phase (1 day). The track of mice in the chamber was recorded for 45 minutes using the VisuTrack system. During the acquisition phase: the mice were injected with saline or METH (2 mg/kg, once a day) and immediately put the mice into the chamber. During the METH withdrawal phase, the mice were administered with saline or CBD (once a day) for 14 days. During the expression phase: the mice were challenged with a low dose of METH (1mg/kg, once a day). The total distance travelled by mice in the chamber was used to assess the behavioral sensitization, and the distance travelled and time spent in the central area, and entries to the central area were utilized to evaluate the anxiety-like behavior of mice^5,6^. Additionally, the detailed protocols of other behavioral testing, including novel object recognition (NOR), elevated plus maze (EPM), social interaction test (SIT), Y maze test, tail suspension test (TST), and forced swimming test (FST), were displayed in the Figure 2A and supplemental methods.

**Novel object recognition (NOR) test**

The NOR test was conducted over two consecutive days in a white plastic box (50cm length × 50cm width × 40cm height)^7^. In the initial test, two identical objects (A and B) were placed into the box. Each mouse was then given 5 minutes to freely explore the box. After two hours, either A or B was randomly replaced with object C, and the mice were given another 5 minutes for exploration. The third test occurred 24 hours after the first test, in which object C was replaced with object D. Object exploration was defined as either sniffing it at a distance of less than 2 cm or touching it with the nose. The VisuTrack system was utilized to record both the exploration time and the number of objects. To evaluate their learning and memory abilities, a recognition index was calculated as follows: new object exploration time / (new object exploration time + old object exploration time) × 100%.

**Elevated plus maze (EPM) test**

EPM test was conducted to evaluate the anxiety-like behavior of mice^5^. The EPM apparatus consisted of a plus-shaped apparatus with two open arms (30cm × 5 cm) and two enclosed arms (30cm × 5cm × 15 cm). The four arms were connected by a central area (5 cm × 5 cm), and the entire apparatus was elevated 70cm off the ground. Each mouse was placed into the central area and allowed to freely explore the apparatus for 5 min. The VisuTrack system was used to measure time spent in the open arm% and the number of entries into open arm%.

**Three-chamber sociability test**

The three-chamber apparatus was a plexiglass box (90 cm × 50 cm × 25 cm), divided into three equal-sized compartments by transparent partitions. At the floor level of each partition, a square opening (5 cm × 5 cm) was located in the center to provide access to each chamber. Two small, round wire cages (diameter 8 cm) were placed in the diagonal corners of the apparatus to enclose the stranger mice. The test was conducted following the procedures outlined in previous studies^8,9^. Each mouse went through three 5-minute sessions, which included: (1) habituation session: two empty cages were placed in the apparatus, and the test mice were allowed to freely explore for 5 minutes; (2) social ability test: a stranger mouse (Stranger 1) was placed in one of the cages, and the test mice were allowed to freely explore for another 5 minutes; (3) social novelty test: a new stranger mouse (Stranger 2) was placed in the empty cage, and the test mice were allowed to freely explore for another 5 minutes. In session (2) and (3), once the test mice were put into the apparatus, the tracks of the mice were immediately recorded using the VisuTrack system. Interaction time was defined as the duration the subject spent sniffing within a 2 cm proximity of the cages^9^. The social ability index was calculated as follows: time spent interacting with Stranger 1 / (time spent interacting with Stranger 1 + time spent in empty interaction) × 100%.

**Y-maze spontaneous alternation**

The spontaneous alternation behavior was used to assess spatial memory in mice using the Y-maze^10^. The Y-maze consists of three arms that are equally spaced apart, forming a Y-shaped configuration. Each arm measures 30 cm in length, 5 cm in width, and 15 cm in height, creating a 120-degree angle. In each trial, the mouse was randomly placed at the end of one arm and allowed to freely explore for 5 minutes. A correct alternation was defined as the mouse not consecutively entering the same arm, following the patterns of ABC, ACB, BAC, BCA, CAB, and CBA. Spontaneous alternation = [(correct alternations)/ (total arm entries − 2)] × 100%^11^.

**Tail suspension test (TST)**

The TST was employed to assess the depressive-like behavior, and the protocol was designed based on a previous study^4^. The mouse’s tail was suspended with adhesive tape in an acoustically and visually isolated chamber, while the tip of its nose was positioned 30 cm above the floor. The mice were suspended for 6 minutes, and the duration of immobility was recorded during the final 5 minutes of the 6-minute test using the VisuTrack system.

**Forced swimming test (FST)**

The FST was conducted as previously described to evaluate the depressive-like behavior^4^. The mouse was placed in a transparent plastic cylinder (diameter 15 cm) that was filled with tap water at 25 ± 1°C to a depth of 30 cm, and the test was performed in both acoustically and visually isolated chambers. Immobility was defined as the animal giving up active struggle and the body floating without writhing. The duration of immobility during the final 5 minutes of the 6-minute test was recorded using VisuTrack software.

**RNA extraction,** **Library construction, and Sequencing**

The mice were sacrificed after behavioral tests, and the NAc tissue was collected from the brain and promptly stored in liquid nitrogen. The total RNA was extracted from the NAc using TRIzol™ reagent (ThermoFisher Scientific, USA) following the manufacturer’s protocol. The quality of RNA samples was assessed using the 5300 Bioanalyzer (Agilent, China), and the concentration was measured using the NanoDrop-2000 (Thermo Fisher Scientific, USA). Small RNA libraries were generated using the QIAseq miRNA Library Kit (Qiagen, Germany) according to the manufacturer’s recommendations. For mRNA + circRNA libraries, the Illumina Stranded Total RNA Prep method was employed with Ribo-Zero Plus for library preparation. Sequencing was conducted by Shanghai Majorbio Bio-pharm Biotechnology Co., Ltd. (Shanghai, China) using an Illumina NovaSeq 6000 platform.

**Identification of circRNAs**

After aligning the clean reads to the reference genome, the junctions of the unmapped reads were identified utilizing a back-splice algorithm. CircRNAs were predicted using CIRI2 ^12^ and Findcirc ^13^. The expression levels of circRNAs were quantified as reads per million mapped reads (RPM), indicating the count of mapped back-splicing junction reads.

**Identification of miRNAs**

To identify miRNAs, all clean mapped tags were aligned with the miRBase22.0 database (http://www.mirbase.org/) to retrieve known miRNAs. The remaining tags were aligned with the Rfam database and Repbase database to exclude ribosomal RNA (rRNA), transfer RNA (tRNA), small nuclear RNA (snRNA), small nucleolar RNA (snoRNA), and other ncRNA and repeats. Subsequently, the unannotated tags were predicted and identified as novel miRNAs using miRdeep2^14^ software, based on their positions in the genome and their hairpin structures.

**Identification of mRNAs**

The raw paired-end reads were trimmed and quality-controlled with default parameters using fastp^15^. The resulting clean reads were then aligned to the reference genome in orientation mode using HISAT2^16^ software. To assemble the mapped reads for each sample, a reference-based approach was performed using StringTie^17^.

**RNA extraction and qRT-PCR**

Total RNA was extracted from the NAc of mice brains using the TransZol Up Plus RNA Kit (TransGen Biotech, China) according to the manufacturer’s protocol. qRT-PCR assays were performed using the CFX96 RT-PCR system (Bio-Rad, USA). The protocols for RNA quantification and the primers used in this study are listed in Supplementary Information. The concentration and quality of RNA were measured by NanoDrop 2000. For circRNAs and mRNA quantification, RNA samples were firstly reversed transcribed into cDNA using Fastking RT Kit (with gDNase) (TIANGEN, China), and relative RNAs expression was quantified using Talent qPCR (SYBR Green) PreMix (TIANGEN, China). For miRNA quantification, RNA samples were firstly reversed transcribed into cDNA by miRNA 1st strand cDNA Synthesis Kit (by tailing A) (Vazyme, China), and relative miRNA expression was quantified using miRNA Universal SYBR qPCR Master Mix (Vazyme, China).

**Western blot**

The total protein of NAc was isolated by treating it with ice-cold RIPA buffer containing protease inhibitors. The protein concentration in each sample was assessed using a BCA protein assay kit (Beyotime, China). Subsequently, the samples were separated by SDS-PAGE and transferred onto a PVDF membrane using a transfer apparatus (BioRed, USA). After blocking the membranes with 5% skim milk at room temperature for 2 hours, the membranes were subsequently incubated overnight at 4 ℃ with the following primary antibodies: mouse anti-β-actin (1:2000, Proteintech, China), rabbit anti-KCNJ5 (1:1000, ThermoFisher Scientific, USA).

1 Liu L, Li J, Wang C, et al*.* Cannabidiol attenuates methamphetamine-induced conditioned place preference in male rats and viability in PC12 cells through the Sigma1R/AKT/GSK3β/CREB signaling pathway. *Am J Drug Alcohol Abuse* 2022; 1–14.

2 Sun L, Song R, Chen Y, et al*.* A selective D3 receptor antagonist YQA14 attenuates methamphetamine-induced behavioral sensitization and conditioned place preference in mice. *Acta Pharmacol Sin* 2016; **37**: 157–165.

3 Fu K, Lin H, Miyamoto Y, et al*.* Pseudoginsenoside-F11 inhibits methamphetamine-induced behaviors by regulating dopaminergic and GABAergic neurons in the nucleus accumbens. *Psychopharmacology* 2016; **233**: 831–840.

4 Ni T, Zhu L, Wang S, et al*.* Medial prefrontal cortex Notch1 signalling mediates methamphetamine-induced psychosis via Hes1-dependent suppression of GABAB1 receptor expression. *Mol Psychiatry* 2022; **27**: 4009–4022.

5 Xiu Y, Kong X-R, Zhang L, et al*.* White Matter Injuries Induced by MK-801 in a Mouse Model of Schizophrenia Based on NMDA Antagonism. *The Anatomical Record* 2014; **297**: 1498–1507.

6 Chen F, Sun J, Chen C, et al*.* Quercetin Mitigates Methamphetamine-Induced Anxiety-Like Behavior Through Ameliorating Mitochondrial Dysfunction and Neuroinflammation. *Front Mol Neurosci* 2022; **15**: 829886.

7 Antunes M, Biala G. The novel object recognition memory: neurobiology, test procedure, and its modifications. *Cogn Process* 2012; **13**: 93–110.

8 Molosh AI, Johnson PL, Spence JP, et al*.* Social learning and amygdala disruptions in Nf1 mice are rescued by blocking p21-activated kinase. *Nat Neurosci* 2014; **17**: 1583–1590.

9 Zhang J-B, Chen L, Lv Z-M, et al*.* Oxytocin is implicated in social memory deficits induced by early sensory deprivation in mice. *Mol Brain* 2016; **9**: 98.

10 Hughes RN. The value of spontaneous alternation behavior (SAB) as a test of retention in pharmacological investigations of memory. *Neurosci Biobehav Rev* 2004; **28**: 497–505.

11 Yin F, Guo H, Cui J, et al*.* The basolateral amygdala regulation of complex cognitive behaviours in the five-choice serial reaction time task. *Psychopharmacology (Berl)* 2019; **236**: 3135–3146.

12 Gao Y, Wang J, Zhao F. CIRI: an efficient and unbiased algorithm for de novo circular RNA identification. *Genome Biol* 2015; **16**: 4.

13 Memczak S, Jens M, Elefsinioti A, et al*.* Circular RNAs are a large class of animal RNAs with regulatory potency. *Nature* 2013; **495**: 333–338.

14 Friedländer MR, Mackowiak SD, Li N, Chen W, Rajewsky N. miRDeep2 accurately identifies known and hundreds of novel microRNA genes in seven animal clades. *Nucleic Acids Res* 2012; **40**: 37–52.

15 Chen S, Zhou Y, Chen Y, Gu J. fastp: an ultra-fast all-in-one FASTQ preprocessor. *Bioinformatics* 2018; **34**: i884–i890.

16 Kim D, Langmead B, Salzberg SL. HISAT: a fast spliced aligner with low memory requirements. *Nat Methods* 2015; **12**: 357–360.

17 Pertea M, Pertea GM, Antonescu CM, Chang T-C, Mendell JT, Salzberg SL. StringTie enables improved reconstruction of a transcriptome from RNA-seq reads. *Nat Biotechnol* 2015; **33**: 290–295.
