# Supplementary material for: Identification of the circRNA–miRNA–mRNA network for treating methamphetamine‐induced relapse and behavioral sensitization with cannabidiol
Source: CNS Neurosci Ther. 2024 May 3;30(5):e14737. doi: 10.1111/cns.14737 (PMC11069028; doi:10.1111/cns.14737)
Supplement: Supplementary file 9 — DataS2 [file CNS-30-e14737-s006.pdf]

Full unedited blot for Figure 6G (n = 5)

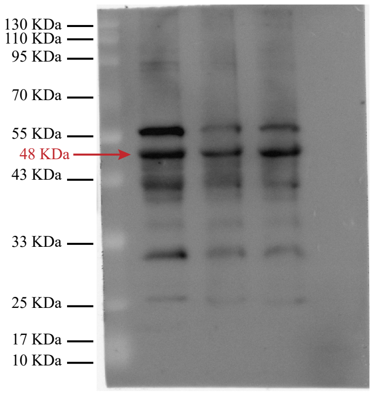

Kcnj5-1

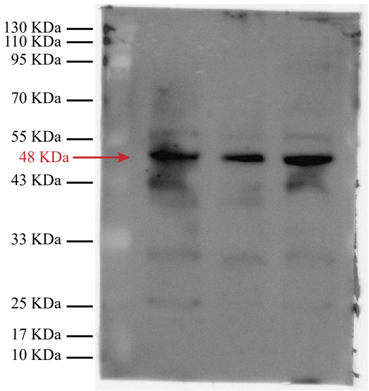

Kcnj5-2

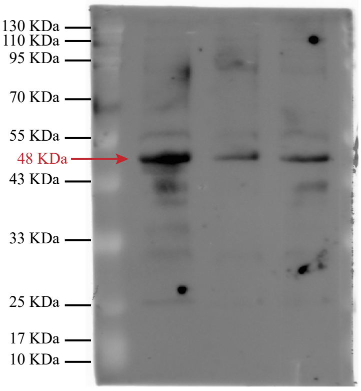

Kcnj5-3

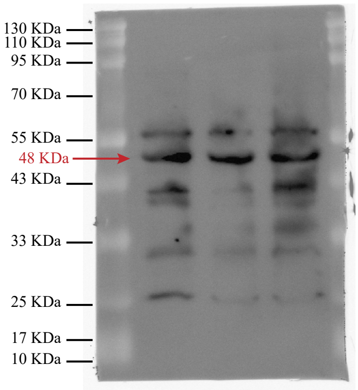

Kcnj5-4

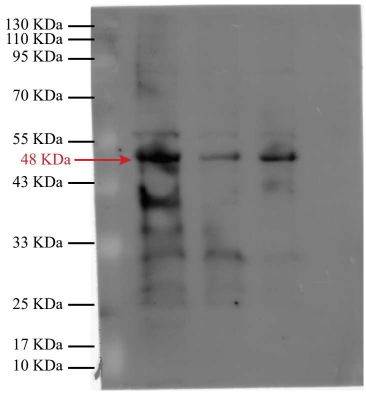

Kcnj5-5

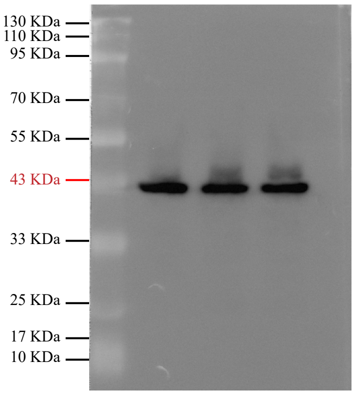

β-actin-1

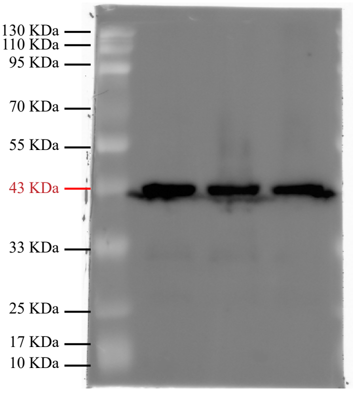

β-actin-2

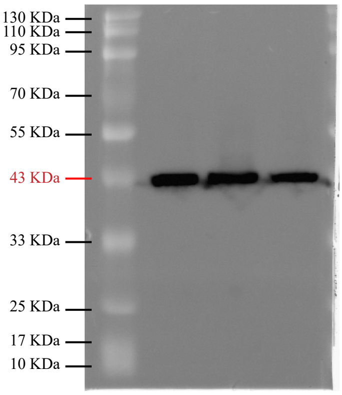

β-actin-3

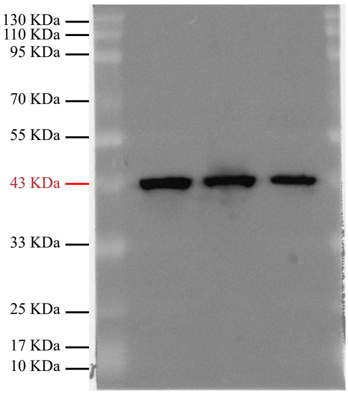

β-actin-4

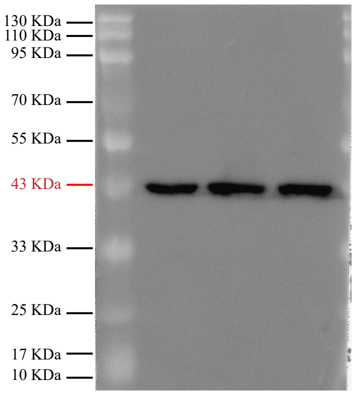

β-actin-5

Full unedited blot for Figure 6N (n = 5)

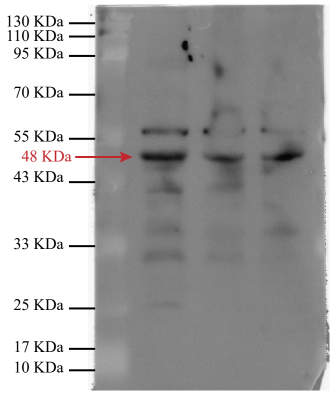

Kcnj5-1

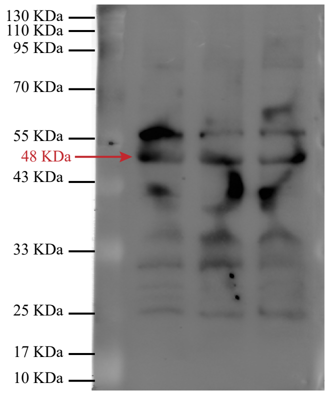

Kcnj5-2

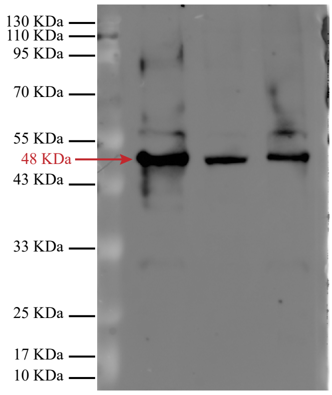

Kcnj5-3

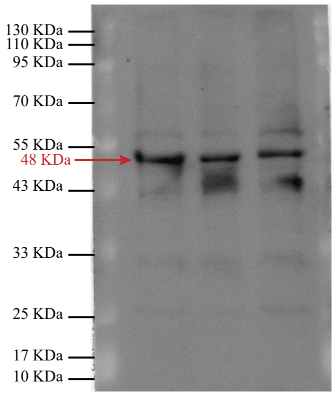

Kcnj5-4

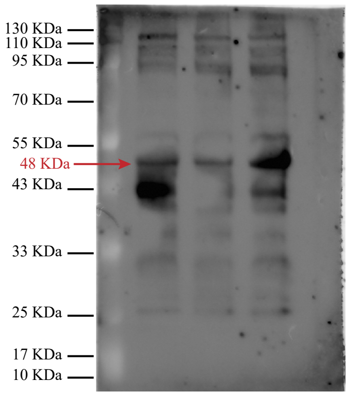

Kcnj5-5

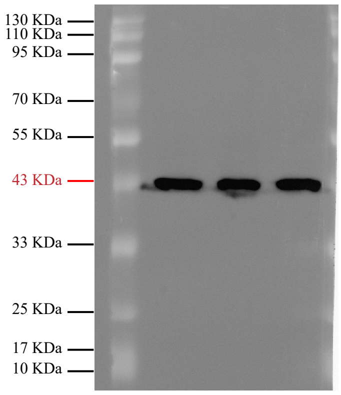

β-actin-1

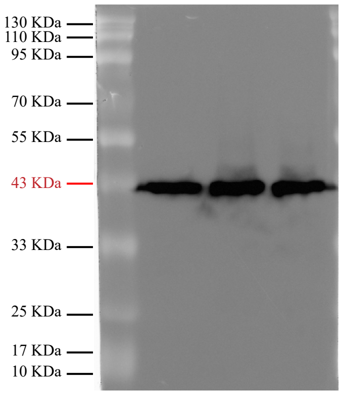

β-actin-2

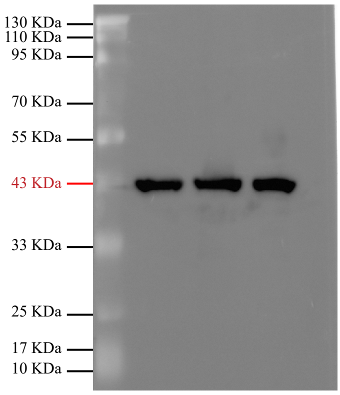

β-actin-3

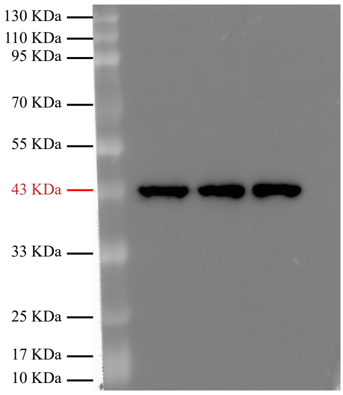

β-actin-4

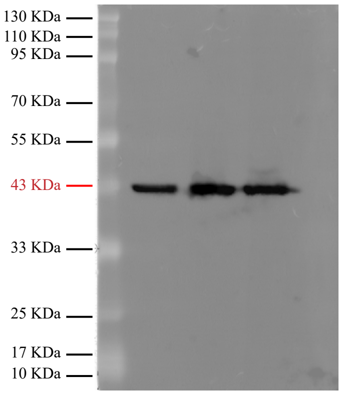

β-actin-5
